# Expression of HOTAIR and MEG3 are negatively associated with *H. pylori* positive status in gastric cancer patients

**DOI:** 10.18632/genesandcancer.219

**Published:** 2022-02-10

**Authors:** Farnaz Amini, Mohammad Khalaj-Kondori, Amin Moqadami, Ali Rajabi

**Affiliations:** ^1^Department of Animal Biology, Faculty of Natural Sciences, University of Tabriz, Tabriz, Iran

**Keywords:** MEG3, HOTAIR, gastric cancer, biomarker, lncRNA

## Abstract

Background: Chronic infection with *Helicobacter pylori* is one of the main causes of gastric cancer (GC). Besides, lncRNAs play crucial roles in cancer pathobiology including GC. Here we aimed to investigate the expression of MEG3 and HOTAIR in gastric cancer tissues and evaluate their association with the *H. pylori* status.

Materials and Methods: One hundred samples were obtained. Total RNA was extracted, cDNA was synthesized and expression of MEG3 and HOTAIR was assessed using qRT-PCR. Association of their expression with *H. pylori* status and other clinicopathological characteristics were investigated. Furthermore, sensitivity and specificity of the MEG3 and HOTAIR expression levels for discrimination of the tumor and non-tumor samples were evaluated by Receiver operating characteristic (ROC) curve analysis.

Results: We observed upregulation of HOTAIR but downregulation of MEG3 in tumor compared to the non-tumor tissues. We also found a significant negative association between their expression levels and *H. pylori* positive status. However, only the expression level of HOTAIR was significantly associated with the size and stage of the tumor (*P* < 0.05). The ROC curve analysis revealed that the expression levels of MEG3 and HOTAIR might discriminate GC tumor and non-tumor tissues.

Conclusions: In conclusion, this study revealed a negative association between H. pylori infection and expression of MEG3 and HOTAIR. The results suggested that the expression level of these lncRNAs might be considered as potential biomarkers for GC.

## INTRODUCTION

According to the GLOBOCAN project of the International Agency for Research on Cancer (IARC), approximately 5.7% of all diagnosed cancer cases belonged to gastric cancer (GC), with 782,685 related deaths in 2018 worldwide [1]. Following the lung and colorectal cancer-related deaths, GC was responsible for 8.2% of all cancer-related deaths and placed in the third most common malignancies in 2018 [1]. It was reported that the incidence of GC was higher in adults aged <50 years, and two- to three-fold higher for men than women [2]. Four subgroups of GC have been identified including chromosomally unstable tumors (50%), Epstein-Barr virus-positive (9%), genomically stable tumors (20%), and microsatellite unstable tumors (22%) [3]. Furthermore, association between *Helicobacter pylori* (*H. pylori*) infection and gastric cancer has also been documented [4, 5]. It was reported that treatment of *H. pylori* infection in patients with early gastric cancer reduced the risk of developing gastric cancer by 50% [6]. A meta-analysis of six randomized trials revealed that treatment of H. pylori infection in general population may prevent developing of gastric cancer by a cancer relative risk of 0.66 [7].

Long noncoding RNAs (lncRNAs) functionally involved in gene expression regulation at chromatin, transcriptional, translational, and post-translational modification levels in cancer progression [4, 5]. Recently, dysregulation of various lncRNAs, such as MIR100HG, BANCR, H19, CASC15, MALAT1, TUSC7, MEG3, and HOTAIR in apoptosis, cell proliferation, migration, metastasis, invasion, and tumorigenicity of GC has been reported by different studies [6–11].

Maternally expressed gene 3 (MEG3) lncRNA is characterized as a tumor suppressor gene and its downregulation has been shown in many cancers, including GC [12]. It has been reported that the expression of MEG3 was lower in GC tissues compared to adjacent normal tissues [10]. This downregulation is mediated by hypermethylation of the MEG3 promoter region which, in turn, provokes GC progression [13, 14]. MEG3 not only regulates the p53 expression but also affects EMT, invasion, and migration of GC via sponging miRNAs including miR-181 and miR-21 by acting as a competing endogenous RNA (ceRNA) [15–17].

LncRNA HOTAIR *(*Hox transcript antisense intergenic RNA), located on chromosome 12q13.13, was first described by Rinn et al. in 2007 [18]. The HOTAIR exerts its function by downregulating the expression of HOXD locus and some other genes. HOTAIR affects expression of multiple genes, especially those involved in the invasion and metastasis [18]. Emerging studies have identified that HOTAIR exerts its function through sponging miRNAs, including miR-34a, miR-331-3p, and miR-217, suggesting its correlation with poor diagnosis of GC [19–21]. Although HOTAIR acts as a tumor oncogene and its overexpression has been indicated in the primary and metastatic GC compared to adjacent non-tumor tissues, still it is regarded as an independent diagnosis biomarker to predict the risk GC and mortality rates in patients with GC [22].

Here we assessed the expression of lncRNAs MEG3 and HOTAIR in GC and analyzed their association with the *H. pylori* status and other clinicopathological characteristics of the patients. Moreover, their biomarker potency was evaluated by the Receiver operating characteristic (ROC) curve analysis.

## RESULTS

### Patients

A total of 100 GC patients were included in the study. Table 1 represents the clinicopathological features of the patients. 68% of the patients were female but 32% male. Regarding age distribution, fifty patients were ≤50 years old and fifty were >50 years old. The majority of patients were positive for *H.pylori* (55%).

**Table 1:** The clinicopathological features of patients with GC

Clinical characteristics	Number
**Age**	
≤50	50
>50	50
**Gender**	
Male	37
Female	63
**Metastasis**	
Yes	43
No	57
**Size**	
≥5 cm	42
<5 cm	58
**Stage**	
I, II	50
III, IV	50
***H. Pylori***	
Positive	55
Negative	45

### Expression of MEG3 and HOTAIR was negatively correlated in GC tissues

**Figure 1:**
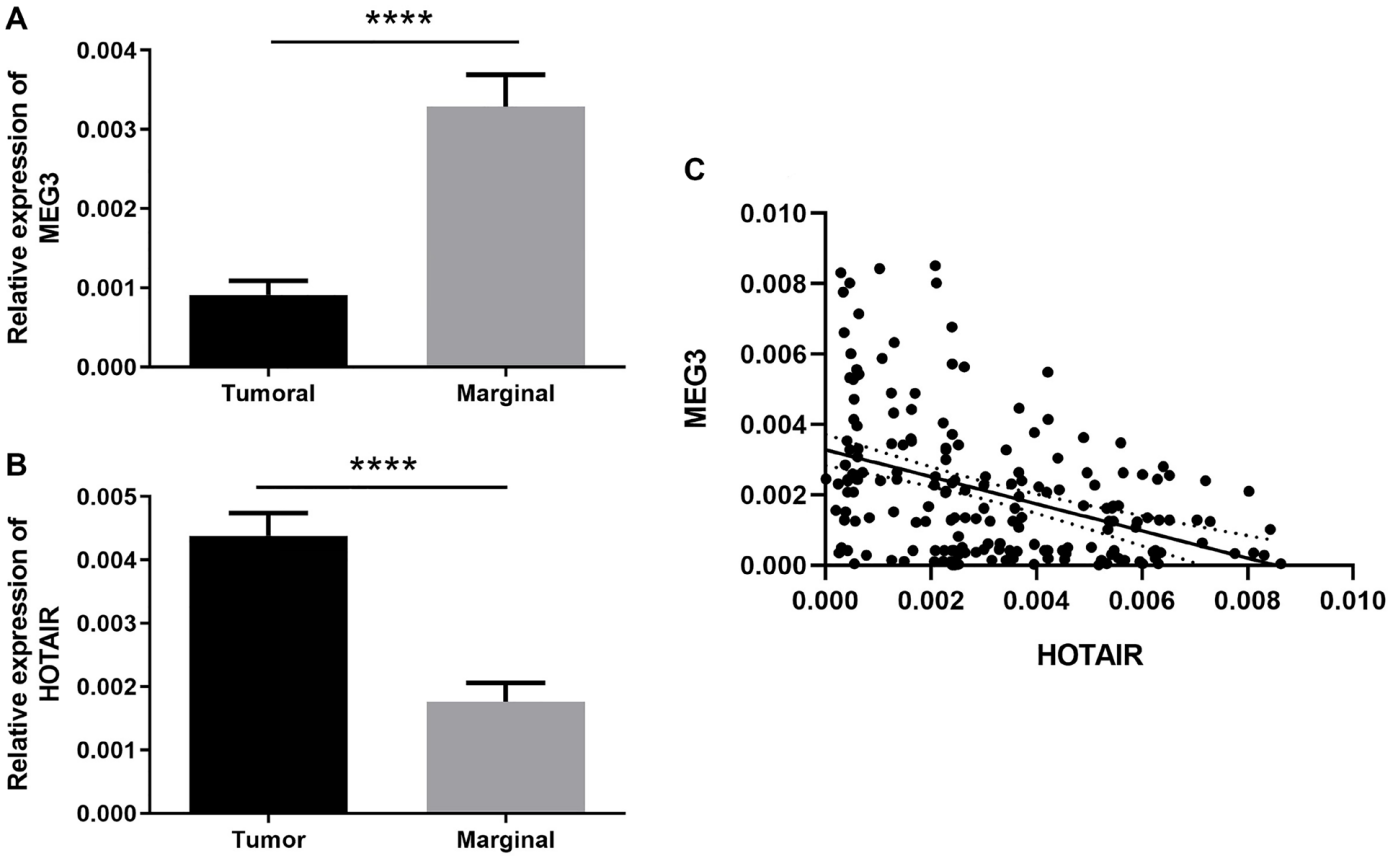
Differences in the expression level of lncRNAs in tumors as compared to the marginal non-tumor tissues at mRNA level represented by bar plot. (**A**) The relative expression of MEG3 in tumor tissues was significantly lower than adjacent non-tumor tissues. (**B**) The relative expression of HOTAIR in tumor tissues was significantly higher than marginal non-tumor tissues. (^****^ represents *P* < 0.0001). (**C**) The correlation between MEG3 and HOTAIR expression level by dot plot. Spearman correlation coefficient is r = −0.409 (95% CI = −0.5211 to −0.2824) which indicates an inverse correlation.

Expression of MEG3 and HOTAIR lncRNAs was quantified by qRT-PCR and compared between tumor and non-tumor tissues. We observed a significant decrease in the MEG3 expression level in tumor tissues compared with the paired non-tumor tissues (*P*-value <0.0001) (Figure 1A). On the other hand, HOTAIR expression showed a significant increase in tumor tissues compared with the paired non-tumor ones (*P*-value <0.0001) (Figure 1B). Finally, we investigated the correlation of MEG3 and HOTAIR expression levels (Figure 1C). The results demonstrated a significant negative correlation (Spearman r = −0.409; *P* <0.0001) indicating an inverse correlation between the expressions of these genes (Figure 1C).

### Expression of MEG3 and HOTAIR were negatively associated with the H-pylori positive status in GC patients

Associations of MEG3 and HOTAIR expression with the clinicopathological characteristics of the patients were also investigated and represented in Table 2. Results showed that MEG3 (*P* = 0.013) and HOTAIR (*P* = 0.001) expression levels were significantly higher in the *H. pylori* negative patients compared with the *H. pylori* positive group, indicating a negative association with the *H. Pylori* infection (Table 2). However, the expression of MEG3 did not show any significant association with the other clinicopathological features. But, we found significant associations between the expression of HOTAIR with the size (*P* = 0.017) and stage (*P* = 0.031) of the tumor. Other patients’ clinicopathological characteristics such as age, gender, and metastasis status did not show any association with the expression level of HOTAIR (Table 2).

**Table 2:** Association between MEG3 and HOTAIR expression with the clinicopathological features in patients with GC

Clinical parameter	No. of cases	Expression means of MEG3 (fold change)	*P*-value	Expression means of HOTAIR (fold change)	*P*-value
**Age**			0.727		0.206
≤50	50	22.15		10.07	
>50	50	17.49		6.04	
**Gender**			0.752		0.400
Male	37	33.92		12.13	
Female	63	10.02		5.22	
**Metastasis**			0.688		0.641
Yes	43	15.09		4.94	
No	57	23.39		10.40	
**Size**			0.331		**0.017**
≥5 cm	42	21.64		8.56	
<5 cm	58	18.21		7.60	
**Stage**			0.593		**0.031**
I, II	50	17.21		9.07	
III, IV	50	22.65		6.96	
***H. Pylori***			**0.013**		**0.001**
Positive	55	17.68		6.66	
Negative	45	24.58		11.16	

### MEG3 and HOTAIR expression levels may discriminate GC tumor and non-tumor samples

**Figure 2:**
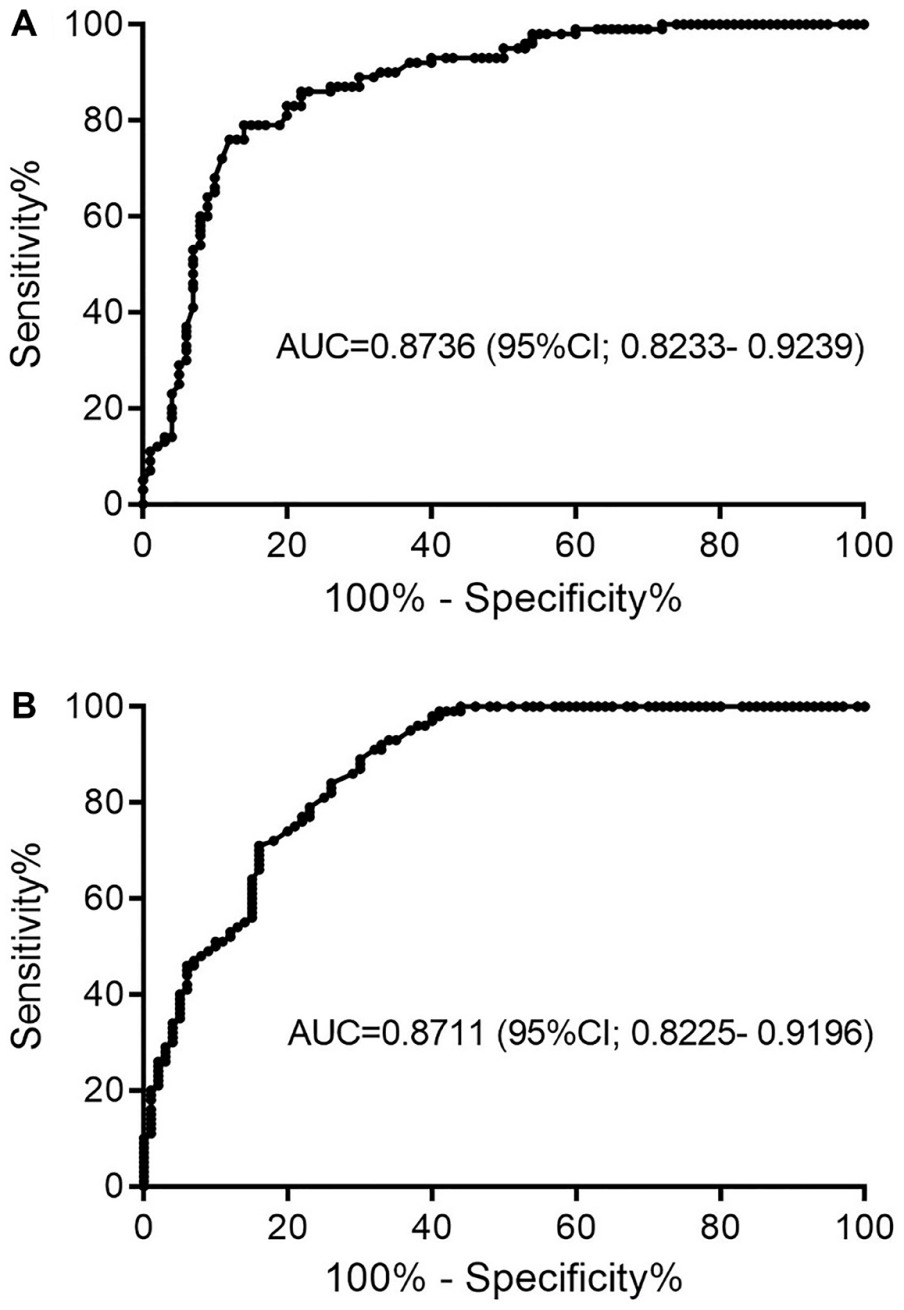
The ROC curve analysis indicating biomarker potency of (**A**) MEG3 and (**B**) HOTAIR. Abbreviation: AUC; Area under the cuve.

To reveal biomarker potency of MEG3 and HOTAIR expression for GC, ROC curve analysis was used (Figure 2). The area under the curve (AUC) of MEG3 was 0.8736 and the AUC of the HOTAIR was 0.8711. Using the cut-off value of 0.0014, the sensitivity and specificity of MEG3 were 79% and 86%, respectively. On the other hand, using the cut-off value of 0.0024, the sensitivity and specificity of HOTAIR were 84% and 74%, respectively (Table 3). These results indicated that both MEG3 and HOTAIR expression levels may effectively discriminate GC tumor from non-tumor samples.

**Table 3:** LncRNAs expression status as diagnostic biomarkers for GC in terms of sensitivity and specificity

Long non-coding RNA	Cutoff value	Sensitivity (%)	Specificity (%)
MEG3	0.0014	79	86
HOTAIR	0.0024	84	74

## DISCUSSION

In the current study, a significantly lower expression of MEG3 was observed in GC tissues as compared to adjacent non-tumor tissues which is in consistent with the tumor suppressor function of this lncRNA. On the other hand, consistent with the oncogenic function of HOTAIR, we observed a significantly higher expression of HOTAIR in GC tissues as compared to adjacent non-tumor tissues. Also, the association of the expressions level of HOTAIR and MEG3 was investigated with regards to *H. pylori* status. The results clearly showed a negative association of the expression levels of the studied lncRNAs and *H. pylori* infection. Furthermore, the diagnostic potential of the two lncRNAs was investigated by ROC curve analysis. The results showed that MEG3 and HOTAIR expression levels could discriminate tumor from non-tumor tissues with specificities 86% and 74%, and sensitivities 79% and 84% respectively. Considering the AUC values 0.8736 and 0.8711 obtained for MEG3 and HOTAIR respectively, it might be concluded that both MEG3 and HOTAIR are potential biomarkers for GC development.

During the past years, MEG3 and HOTAIR expression has been investigated in various cancer types; however, the precise molecular mechanism by which these lncRNAs promote tumorigenesis remains unclear. Regarding the role of MEG3 as a tumor suppressor gene, a study conducted by Guo et al. demonstrated the downregulation of MEG3 in endometrial carcinoma [23]. MEG3 positively was correlated with p53 expression and a decrease in MEG3 expression level was resulted in downregulation of p53 and apoptosis suppression [10, 24]. Besides, MEG3 functions through repressing notch1 and PI3K/m-TOR signaling pathways [25]. Wei et al. reported downregulation of MEG3 in GC tissues compared to adjacent normal tissues and demonstrated a negative correlation between MEG3 expression and the tumor size and TNM stage. Based on these findings, they proposed MEG3 as a potential diagnostic biomarker for GC [10]. The same suggestion was concluded by Sun et al. [13]. Another study evaluated the MEG3 expression and showed that its promoter region hypermethylation leads to downregulation of MEG3 in GC tissues compared to normal tissues [14]. Besides, they demonstrated the negative expression of MEG3 with TNM stage and tumor size. Furthermore, they suggested that MEG3 regulates the apoptosis of GC by targeting of miR-21 [14]. Another study showed downregulation of MEG3 in GC tissues by sponging miR-181 and its contribution to the GC invasion [15]. All these reports are in line with our findings.

It has been reported that HOTAIR contributes to epigenome regulation and chromatin remodeling [24]. Polycomb proteins act as an inhibitor of transcription of several genes, which contribute to cell differentiation [26, 27]. These proteins exist in polycomb repressive complex (PRC) 1 and PRC2. PRC2 regulates the H3K27 methylation and involves in gene silencing [28]. In fact, HOTAIR binds and guides the PRC2, where PRC2 exerts the function of gene expression silencing. Furthermore, p21WAF1/CIP1is a gene that is repressed by HOTAIR, resulting in dysfunction of p53-induced growth arrest and apoptosis [29]. Xiao et al. suggested that HOTAIR regulated proliferation and metastasis of GC through SDF-1/CXCR4 and RhoA signaling pathways by sponging miR-126 [22]. They observed overexpression of HOTAIR in GC and demonstrated that HOTAIR reduces the miR-126 expression, leading to GC invasiveness [22]. HOTAIR was associated with clinicopathological features of GC patients, including tumor size and tumor stage [20, 30], suggesting its potential as a diagnostic biomarker in GC [30]. Our results are in consistent with these reports.

Recently, a research group evaluating the expression of HOTAIR in 49 GC tissues and their adjacent non-tumor tissues has reported that expression level of HOTAIR is not related to the proportion of H. pylori infection (*P* = 0.30) [31]. In this work they showed that HOTAIR interacts with miR-148b and DNA methyltransferase 1 (DNMT1). The DNMT1 promotes methylation of a well-known tumor suppressor gene, Protocadherin 10 (PCDH10), which contributes to the progression of GC [31]. Similarly, a previous study investigating the relation between mir-141 and MEG3 in GC tissues in comparison with adjacent non-tumor tissues has demonstrated no significant changes in MEG3 expression in *H. pylori* positive samples compared to *H. pylori* negative samples (*P* > 0.05) [32]. In a study conducted in 2019, a study group has assessed the association of HOTAIR expression level and H. pylori infection [33]. Opposite to our finding, the results of this study have indicated a significant higher HOTAIR expression in *H. pylori* positive patients (20.8%) compared to *H. pylori* negative patients (2.9%) with *P*-value of 0.017 [33]. This discrepancy might be due to differences in the populations genetic background and reflects the effect of complex interactions between lncRNAs, miRNAs and mRNAs governing cancer cell behavior [34].

We also investigated the correlation of HOTAIR and MEG3 expression GC samples. Our results revealed a negative correlation between HOTAIR and MEG3 expressions, meaning that a higher expression level HOTAIR might lead to the downregulation of MEG3. This finding is in line with other reports and confirms the functional link of HOTAIR and MEG3 in GC pathology. As reported by Bian et al., upregulation of HOTAIR occupies the PRC2 and drastically increases the H3K27me3 at the promoter region of MEG3, leading to epigenetically silencing of MEG3 in the hepatic stellate cells [35]. Finally, MEG3 functions through p53/MDM2 signaling pathways and downregulates p53, leading to GC development [35].

## MATERIALS AND METHODS

### Patients and samples

One hundred gastric tumor and adjacent non-tumor tissues were obtained from Noor-E-Nejat hospital, Tabriz, Iran. Written informed consent was acquired from all participants and the study was approved by the Medical Ethical Committee of the University of Tabriz (IR. TABRIZU. REC. 1398.015). An expert pathologist assessed, characterized, and validated the histopathology features of the tissue’s samples.

### RNA isolation, cDNA synthesis and qRT-PCR

Total RNA was extracted from tumor and non-tumor tissues using TRIZOL reagent (Invitrogen, Massachusetts, USA) according to the company’s protocol. DNaseI (GeneAll, Seoul, Korea) was utilized to eliminate any DNA contamination. NanoDrop (Thermo Fisher scientific Nanodrop 2000, CA, USA) was used to assess the quantity and quality of RNA samples followed by 2% (v/w) agarose gel electrophoresis.

The PrimeScript™ 1st Strand cDNA Synthesis Kit (TaKaRa, Kusatsu, Japan) was used to synthesis the cDNA according to the manufacturer’s protocol. Approximately 100 ng of cDNA was used for amplification of lncRNAs MEG3 and HOTAIR using LightCycler^®^ 96 Real-Time PCR (Roche Molecular Systems, Inc., Pleasanton, CA, USA) with SYBR Green Master Mix (2x) (Amplicon, Odense, Denmark). The primer pairs utilized for amplification of MEG3, HOTAIR, and β-actin (as internal control) were as followings: MEG3; F: 5′-CTGCCCATCTACACCTCACG-3′, R: 5′-CTCTCCGCCGTCTGCGCTAGGGGCT-3′, HOTAIR; F: 5′-GGCAGCACAGAGCAACTCTA-3′, R: 5′-GAGTGCAAAGTCCCGTTTG-3′, and β-actin; F: 5′-AGAGCTACGAGCTGCCTGAC-3′, R: 5′-AGCACTGTCTTGGCGTACAG-3′. The relative expressions were calculated by the formula 2^−ΔCt^. All reactions were run in duplicate format.

### ROC curve analysis

The potential of the MEG3 and HOTAIR as diagnostic biomarkers in GC was assessed by the receiver operating characteristic curve analysis. The ROC curve plots the true positive rate (TPR) against the false positive rate (FPR) at various threshold settings. Thus, the sensitivity and specificity of the markers can be determined at specific threshold values. Also, the area under the curve (AUC) was identified to describe the variation of the sensitivity and specificity of the lncRNAs.

### Statistical analysis

The expression of MEG3 and HOTAIR in tumors and non-tumor samples were compared with Mann-Whitney statistical test. The association between MEG3 and HOTAIR expression levels and the clinicopathological features was investigated using student’s *t*-test and one-way ANOVA. *T*-test was employed in the case of normal distribution, otherwise, the Mann-Whitney test was used. The correlation between MEG3 and HOTAIR expression levels was analyzed by Spearman correlation test. All statistical analyses were performed by GraphPad Prism v.8.4.3 and *P*-values <0.05 were considered as significant.

## CONCLUSIONS

The expression of MEG3 and HOTAIR was negatively associated with the *H. pylori* positive status. MEG3 was downregulated but HOTAIR was upregulated in the GC tissues. The expression of HOTAIR was associated with the size and stage of tumor. Furthermore, MEG3 and HOTAIR expression levels might be considered as potential diagnostic biomarkers for GC.
